# Microbiota-based model improves the sensitivity of fecal immunochemical test for detecting colonic lesions

**DOI:** 10.1186/s13073-016-0290-3

**Published:** 2016-04-06

**Authors:** Nielson T. Baxter, Mack T. Ruffin, Mary A. M. Rogers, Patrick D. Schloss

**Affiliations:** Department of Microbiology and Immunology, University of Michigan, Ann Arbor, MI USA; Department of Family Medicine, University of Michigan, Ann Arbor, MI USA; Department of Internal Medicine, University of Michigan, Ann Arbor, MI USA

## Abstract

**Background:**

Colorectal cancer (CRC) is the second leading cause of death among cancers in the United States. Although individuals diagnosed early have a greater than 90 % chance of survival, more than one-third of individuals do not adhere to screening recommendations partly because the standard diagnostics, colonoscopy and sigmoidoscopy, are expensive and invasive. Thus, there is a great need to improve the sensitivity of non-invasive tests to detect early stage cancers and adenomas. Numerous studies have identified shifts in the composition of the gut microbiota associated with the progression of CRC, suggesting that the gut microbiota may represent a reservoir of biomarkers that would complement existing non-invasive methods such as the widely used fecal immunochemical test (FIT).

**Methods:**

We sequenced the 16S rRNA genes from the stool samples of 490 patients. We used the relative abundances of the bacterial populations within each sample to develop a random forest classification model that detects colonic lesions using the relative abundance of gut microbiota and the concentration of hemoglobin in stool.

**Results:**

The microbiota-based random forest model detected 91.7 % of cancers and 45.5 % of adenomas while FIT alone detected 75.0 % and 15.7 %, respectively. Of the colonic lesions missed by FIT, the model detected 70.0 % of cancers and 37.7 % of adenomas. We confirmed known associations of *Porphyromonas assaccharolytica*, *Peptostreptococcus stomatis*, *Parvimonas micra*, and *Fusobacterium nucleatum* with CRC. Yet, we found that the loss of potentially beneficial organisms, such as members of the Lachnospiraceae, was more predictive for identifying patients with adenomas when used in combination with FIT.

**Conclusions:**

These findings demonstrate the potential for microbiota analysis to complement existing screening methods to improve detection of colonic lesions.

**Electronic supplementary material:**

The online version of this article (doi:10.1186/s13073-016-0290-3) contains supplementary material, which is available to authorized users.

## Background

Colorectal cancer (CRC) mortality has steadily declined in recent decades, due in large part to increased screening [1]. Yet current screening tests, the fecal immunochemical test (FIT) and the multitarget DNA test, have a sensitivity of 7.6 % and 17.2 %, respectively, for detecting non-advanced adenoma – just the type of early lesion that screening is meant to identify [2]. Although structural exams including colonoscopy and sigmoidoscopy are able to detect both adenomas and carcinomas, the high cost and invasive nature are barriers for many people. Fear, discomfort, and embarrassment are among the most cited reasons patients choose to forego CRC screening [3]. Likewise, the large disparity in screening rates between those with and without health insurance highlights the need for inexpensive screening methods [1, 4, 5]. Unfortunately cheaper, less invasive stool-based tests like guaic fecal occult blood test (gFOBT) and FIT are unable to reliably detect adenomas [6]. The newly introduced stool DNA panel has improved accuracy compared to FIT, but is still limited in its ability to accurately detect adenomas [2]. Thus there is need for novel screening methods that are inexpensive and capable of detecting both cancer and adenomas.

The gut microbiota, the collection of microorganisms that inhabit the gastrointestinal tract, are one potential source of biomarkers for detecting colonic lesions. Numerous studies have observed alterations in the gut bacterial communities of patients with CRC [7–12]. Experiments in animal models have demonstrated that such alterations have the potential to accelerate tumorigenesis [13]. Furthermore, several members of the gut microbiota have been shown to potentiate both the development and progression of CRC by a variety of mechanisms [14–16]. Although each of these organisms may play a role in certain cases of CRC, none of them is present in every case. Therefore we postulate that no one organism is an effective biomarker on its own and that focusing on a single bacterial population excludes the potential that the microbial etiology of the disease is actually polymicrobial.

Two recent studies used statistical models that take into account the abundances of multiple bacterial species and the results of gFOBT to distinguish healthy individuals from those with CRC [17, 18]. The analysis by Zackular et al. [17] used samples from a limited number of participants (n = 30 normal, 30 adenoma, and 30 carcinoma), while that of Zeller et al. [18] had a larger cohort from multiple clinical sites (n = 156 and n = 335). A shortcoming of the Zeller study was the pooling of participants with non-advanced adenomas with control participants as well as the exclusion of participants with advanced adenomas. A limitation of both studies was that they relied on gFOBT rather than FIT to detect hemoglobin in stool. FIT provides a quantitative measure of hemoglobin concentrations and has largely replaced gFOBT clinically because of its improved sensitivity. Regardless of their weaknesses, these studies demonstrated the feasibility of using microbiome data to identify participants with colonic lesions.

In the present study, we demonstrate the potential for microbiota analysis to complement FIT for improved detection of colonic lesions, especially adenomas. We utilized the random forest algorithm, which is a decision tree-based machine learning algorithm for classification that accounts for non-linear data and interactions among features and includes an internal cross-validation to prevent overfitting [19]. With this method we identified bacterial populations that could distinguish healthy individuals from those with adenomas or carcinomas. In doing so, we confirmed previously observed associations of certain bacterial taxa with CRC. Many lesions detected using the microbiota were distinct from those detected by FIT, suggesting the microbiota could complement FIT to improve sensitivity. By incorporating data on hemoglobin and bacterial abundances into a single model (labeled the multitarget microbiota test or MMT), we were able to improve the sensitivity for adenomas and cancer compared to FIT alone.

## Methods

### Study design/patient sampling

Eligible patients for this study were aged at least 18 years, willing to sign informed consent, able to tolerate removal of 58 mL of blood, and willing to collect a stool sample. Patient age at the time of enrollment was in the range of 29–89 years with a median of 60 years. All patients were asymptomatic and were excluded if they had undergone surgery, radiation, or chemotherapy for current CRC prior to baseline samples or had inflammatory bowel disease, known hereditary non-polyposis CRC, or familial adenomatous polyposis. Colonoscopies were performed and fecal samples were collected from participants in four locations: Toronto (ON, Canada), Boston (MA, USA), Houston (TX, USA), and Ann Arbor (MI, USA). Patient diagnoses were determined by colonoscopic examination and histopathological review of any biopsies taken. Patients with an adenoma greater than 1 cm, more than three adenomas of any size, or an adenoma with villous histology were classified as advanced adenoma. Whole evacuated stool was collected from each patient either prior to colonoscopy preparation or 1–2 weeks after colonoscopy. This has been shown to be sufficient time for the microbiota to recover from colonoscopy preparation [20]. Stool samples were packed in ice, shipped to a processing center via next day delivery, and stored at –80 °C. The University of Michigan Institutional Review Board approved this study, and all participants provided informed consent. This study conformed to the guidelines of the Helsinki Declaration.

### Fecal immunochemical tests

Fecal material for FIT was collected from frozen stool aliquots using OC FIT-CHEK sampling bottles (Polymedco Inc.) and processed using an OC-Auto Micro 80 automated system (Polymedco Inc.). Hemoglobin concentrations were used for generating receiver operating characteristic (ROC) curves for FIT and for building the MMT.

### 16S rRNA gene sequencing

DNA was extracted from approximately 50 mg of fecal material from each participant using the PowerSoil-htp 96 Well Soil DNA isolation kit (MO BIO Laboratories) and an epMotion 5075 automated pipetting system (Eppendorf). The V4 region of the bacterial 16S rRNA gene was amplified using custom barcoded primers and sequenced as described previously using an Illumina MiSeq sequencer [21]. The 490 samples were divided into three sequencing runs to increase the per sample sequencing depth. Although the same percentage of samples from the three groups were represented on each sequencing run, samples were randomly assigned to the sequencing runs to avoid confounding our analysis based on diagnosis or demographics.

### Sequence curation

The 16S rRNA gene sequences were curated using the mothur software package (v1.36), as described previously [21, 22]. Briefly, paired-end reads were merged into contigs, screened for quality, aligned to SILVA 16S rRNA sequence database, and screened for chimeras. Sequences were classified using a naive Bayesian classifier trained against a 16S rRNA gene training set provided by the Ribosomal Database Project (RDP) [23]. Curated sequences were clustered into operational taxonomic units (OTUs) using a 97 % similarity cutoff with the average neighbor clustering algorithm. Species-level classifications for OTUs of interest were determined by blasting the predominant sequences within each OTU to the NCBI 16S rRNA database. The putative species was only reported for OTUs with greater than 99 % sequence identity to a single species in the database; otherwise the consensus RDP classification was used. The number of sequences in each sample was rarefied to 10,000 per sample to minimize the effects of uneven sampling. Only the 335 OTUs present in at least 5 % of samples were included in the feature selection for the random forest models.

### Statistical methods

All statistical analyses were performed using R (v.3.2.0). Random Forest models were generated using the AUCRF package [24]. All ROC curves presented for random forest models are based on the out-of-bag (OOB) error rates. For each model, leave-one-out and 10-fold cross-validations were performed to further estimate the generalization error of the model. The AUC of ROC curves were compared using the method described by DeLong et al. [25]. The optimal cutoff for the MMT was determined using Youden’s *J* statistic [26]. This cutoff was determined using the ROC curve for differentiating cancer from normal. Comparisons of sensitivities of FIT and the MMT at the same specificity were performed using the method developed by Pepe et al. with 1000 bootsrap replicates [27]. All of the aforementioned statistics for analyzing ROC curves were performed using the pROC package in R [28]. To control for diagnosis while testing the effects of sex on the microbiome we used PERMANOVA as implemented in the adonis function in the vegan R package [29].

## Results

### Complementary detection of lesions by FIT and the microbiota

**Fig. 1:**
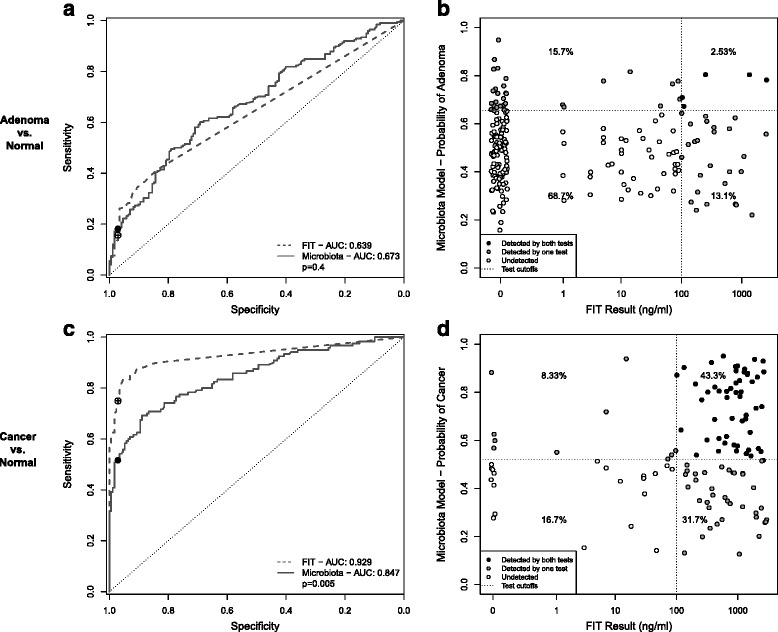
Microbiota-based models can complement FIT. **a, c** ROC curves for distinguishing healthy patients from those with adenoma (**a**) or cancer (**c**) based on FIT or a microbiota-based random forest model. *Open circles* show the sensitivity and specifity of FIT with a 100 ng/mL cutoff. *Black points* show the sensitivity and specificity of the microbiota-based models at the same specificity as FIT. **b**, **d** Results of FIT and a microbiota-based model for each adenoma (**b**) or cancer (**d**) sample. *Dotted lines* represent the cutoffs for each test. Points are shaded based on whether the lesion was detected by both tests (*black*), one of the two tests (*gray*), or neither test (*white*)

We characterized the bacterial communities of stool samples from 490 patients using 16S rRNA gene sequencing. Among these patients, 120 had CRC, 198 had adenomas, and 172 had no colonic lesions. In addition to characterizing the bacterial community, we tested each sample for the concentration of hemoglobin using FIT. With these data, we compared the ability to detect lesions using FIT to using a microbiota-based model. First, we developed a random forest classification model for differentiating healthy individuals from those with adenomas based on the relative abundance of bacterial populations in stool. We determined the optimal model using the AUC-RF algorithm for maximizing the area under the curve (AUC) of the ROC curve for a random forest model [24]. The optimal model utilized 22 bacterial populations (Additional file 1: Figure S1A). The vast majority of OTUs in the model (17 out of 22) belonged to the order Clostridales, four were associated with the genus *Bacteroides*, and one OTU was unclassified at the phylum level (Additional file 1: Figure S1B). The AUC for this and subsequent random forest models were generated based on the OOB probabilities for each sample. Additional leave-one-out and 10-fold cross validations showed no significant difference in AUC compared to the OOB AUC (Additional file 2: Figure S2A). The AUC for the microbiota model (0.673) was significantly different from a random assignment (*p* <0.001), but not significantly different from that of FIT (FIT AUC:0.639, *p* >0.05, Fig. 1a). At the 100 ng/mL cutoff, FIT detected 15.7 % of adenomas with a specificity of 97.1 %. Setting the microbiota model to the same 97.1 % specificity resulted in 18.2 % sensitivity for adenomas. When comparing the results of the tests for each sample, only 2.5 % of adenomas were detected by both tests, while 28.8 % were detected by only one of the two tests (Fig. 1b). Thus, the two tests detected small but distinct subsets of adenomas.

Next we generated a random forest model for differentiating normal individuals from those with cancer using the relative abundance of 34 bacterial populations (Additional file 3: Figure S3A and S3B). Consistent with previous observations, the bacteria most strongly associated with CRC belonged to taxa commonly associated with periodontal disease [18, 30, 31]. These include OTUs associated with *Pophyromonas assaccharolytica* (OTU105), *Fusobacterium nucleatum* (OTU264), *Parvimonas micra* (OTU281), *Peptostreptococcus stomatis* (OTU310), *Gemella spp.* (OTU356), and an unclassified *Prevotella* (OTU57) (Additional file 3: Figure S3C). The ROC curve for the model had an AUC of 0.847, which was similar to AUCs reported for other microbiota-based models for CRC [17, 18]. The AUC of this model was significantly better than a random assignment (*p* <0.001), but was significantly lower than that of FIT (FIT AUC:0.929, *p* = 0.005, Fig. 1c). As with the adenoma versus normal model, we confirmed the OOB AUC with leave-one-out cross validation and 100 iterations of 10-fold cross validation (Additional file 2: Figure S2B). At the manufacturer’s recommended cutoff of 100 ng/mL, FIT detected 75.0 % of cancers with a specificity of 97.1 %. At the same specificity, the microbiota model detected 51.7 % of cancers. Although more cancers were detected by FIT, the microbiota model was able to detect 33.3 % of cancers missed by FIT (Fig. 1d).

### MMT for colonic lesions

Many of the adenomas and some of the carcinomas were detected by the microbiota models, but not FIT, suggesting that the two screening methods could complement each other if combined into a single test. Based on these observations, we developed a random forest model using both the microbiota and FIT that would differentiate normal individuals from those with any type of colonic lesion (i.e. adenoma or carcinoma). The optimal model, referred to as the MMT, used the relative abundances of 23 OTUs and the concentration of hemoglobin as determined by FIT. Of those OTUs, 16 were members of the Firmicutes phylum, including three from the Ruminococcaceae family and 10 from the Lachnospiraceae family (Additional file 4: Figure S4). Three OTUs were associated with the genus *Bacteroides*. The remaining OTUs were associated with *Porphyromonas*, *Parabacteroides*, *Collinsella*, and Enterobacteriaceae. The OTU associated with *Porphyromonas* was most closely related to *Porphyromonas asaccharolytica*, which has been previously shown to be predictive of CRC [17, 18, 32]. Interestingly the majority of OTUs used in the model, especially the Lachnospiraceae, were enriched in normal patients (Additional file 4: Figure S4), suggesting that a loss of beneficial organisms in addition to the emergence of pathogens may be indicative of CRC development. As with the previous random forest models we performed leave-one-out cross validation and 100 iterations of 10-fold cross-validation and found no difference in AUC compared to the OOB estimates (Additional file 5: Figure S5).

### Comparing MMT to FIT

**Fig. 2:**
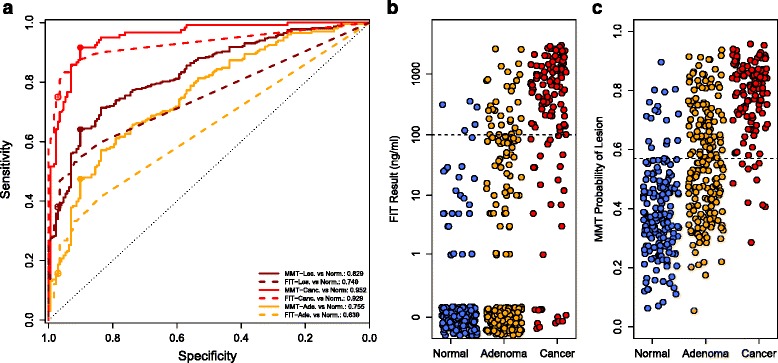
Comparing MMT to FIT. **a** ROC curves for the MMT (*solid lines*) or FIT (*dashed lines*) for distinguishing normal from any lesion (*dark red*), normal from cancer (*red*), and normal from adenoma (*orange*). *Filled dots* show the sensitivity and specificity of the MMT at the optimal cutoff (0.57). *Open dots* show the sensitivity and specificity of FIT at the 100 ng/mL cutoff. **b**, **c** Stripcharts showing the results for FIT (**b**) and the MMT (**c**). *Dashed lines* show the cutoff for each test. Points with a FIT result of 0 are jittered to improve visibility

To determine whether microbiota sequence data could be used to complement FIT, we compared the performance of the MMT to FIT. For differentiating any lesions from normal, the AUC for the MMT was significantly higher than FIT (MMT AUC: 0.829, FIT AUC: 0.749, *p* <0.001, Fig. 2a). Subdividing the lesions, detecting adenomas by the MMT (AUC: 0.755) was significantly better than FIT (AUC: 0.639, *p* <0.001), but not for differentiating cancer from normal (MMT AUC: 0.952, FIT AUC: 0.929, *p* = 0.09). To generate a categorical prediction from the MMT, we determined the model’s optimal threshold for detecting cancer (0.57 probability of a lesion) using Youden’s J statisitc [26]. Samples scoring above this cutoff were classified as lesions, and those below the cutoff were classified as normal. We then compared the sensitivity and specificity of the MMT to those of FIT using a threshold of 100 ng/mL of hemoglobin. At these cutoffs, the MMT detected 91.7 % of cancers and 45.5 % of adenomas compared to 75.0 % and 15.7 % for FIT (Table 1, Fig. 2b, c). When adenomas and cancers were pooled together, the MMT detected 62.9 % of lesions, while FIT only detected 38.1 %. However, the increased sensitivity of the MMT was accompanied by a decrease in specificity (90.1 %) compared to FIT (97.1 %).

**Table 1:** Sensitivities and specificities for FIT and MMT. The 95 % confidence intervals were computed with 2000 stratified bootstrap replicates

Diagnosis		Fecal immunochemical test		Multitarget microbiota test	
		True positives	Sensitivity (95 % CI)	True positives	Sensitivity (95 % CI)
Cancer	n = 120	90	75.0 (67.5–82.5)	110	91.7 (86.7–95.8)
Adenoma	n = 198	31	15.7 (10.6–20.7)	90	45.5 (38.4–52.5)
Any lesions	n = 318	121	38.1 (32.7–43.4)	200	62.9 (57.2–67.9)
		True negatives	Specificity (95 % CI)	True negatives	Specificity (95 % CI)
Normal	n = 172	167	97.1 (94.2–99.4)	155	90.1 (85.5–94.2)

**Fig. 3:**
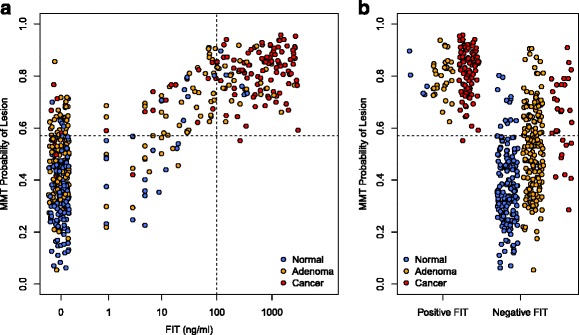
Relationship between FIT and MMT for each sample. **a** Scatterplot of MMT and FIT results for each sample. *Dashed lines* show the cutoff for each test. Points with a FIT result of 0 are jittered to improve visibility. **b** Stripchart of MMT results for samples separated by binary FIT result

To better understand the relationship between the MMT and FIT, we compared the results of the two tests for each sample (Fig. 3a). All but one of the samples that tested positive by FIT also tested positive by the MMT. However, the MMT was able to detect 70.0 % of cancers and 37.7 % of adenomas that FIT had failed to detect, while maintaining a specificity of 92.8 % (Fig. 3b). This result demonstrated that incorporation of data from a participant’s microbiota could complement FIT to improve its sensitivity.

**Fig. 4:**
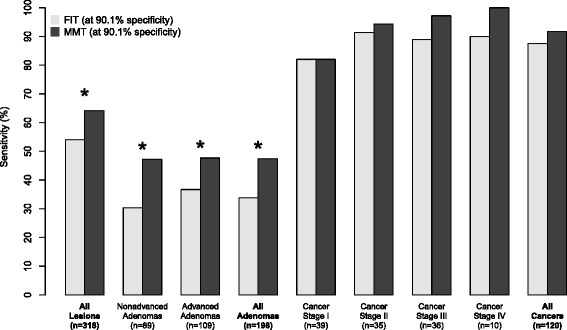
Sensitivities for FIT and MMT for each stage of tumor development with matching specificities. The cutoff for FIT was reduced to 7 ng/mL to match the specificity of the MMT. Sensitivities were compared using the method proposed by Pepe et al. (* = *p* <0.05, 1000 bootstrap replicates)

To make a fairer comparison of the sensitivities of these two tests, we reduced the cutoff for FIT to 7 ng/mL to match the 90.1 % specificity of the MMT. At the lower cutoff for FIT there was no significant difference in sensitivity for cancer between the two tests (*p* = 0.2), but the MMT remained significantly more sensitive for detecting adenomas (*p* = 0.02) and all lesions grouped together (*p* = 0.04, Fig. 4).

The purpose of screening is to identify asymptomatic individuals with early stage disease (i.e. true positives). Therefore, we estimated the number of true positives captured through FIT and MMT in the recommended screening population in the United States (adults aged 50–75 years). The prevalence of lesions in an average-risk population was obtained through a previously published meta-analysis [33]. Based on sensitivities of FIT and MMT in our dataset, we estimate that MMT would detect approximately 40 thousand additional cancers, 1.3 million additional advanced adenomas, and 5.1 million additional non-advanced adenomas compared to using FIT (Table 2). Thus the improved sensitivity of the MMT would increase the total number of true positives identified in the recommended screening population of the United States by approximately 6.5 million. However, due to the lower specificity of MMT, it would also result in an estimated 4.3 million additional false positives compared to FIT. Further studies would be needed to determine whether detection of 6.5 million additional lesions (mostly non-advanced adenomas) would outweigh the added cost of 4.3 million additional false positives.

**Table 2:** Estimated number of true positives detected in average risk population. Number of true positives identified through FIT and MMT in the United States in adults aged 50–75 years, based on published estimates of CRC prevalence. The sensitivities for FIT (100 ng/mL cutoff) on advanced and non-advanced adenomas were 19.3 % and 11.2 %, respectively

Condition	Prevalence	Number of persons, aged 50–75 years, with condition^a^	True positives identified by FIT	True positives identified by MMT
Cancer	0.3 %	241,483	181,112	221,359
Advanced adenoma	5.7 %	4,588,174	883,960	2,188,854
Non-advanced adenoma	17.7 %	14,247,488	1,600,841	6,723,534

^a^Number of persons in the United States in 2010 aged 50–75 years was 80,494,283

### Effect of patient characteristics on model performance

Previous studies have identified differences in diagnostic test performance for certain demographic groups or for people taking certain medications [34–36]. Therefore we tested whether the MMT performance differed between patient populations. We found no difference in model performance according to age, BMI, NSAID usage, diabetes, smoking, or previous history of polyps (all *p* > 0.05). However, the model was significantly better at differentiating normal from lesion for women than for men (*p* = 0.02; Additional file 6: Figure S6). For women the model detected 63.6 % of lesions with a specificity of 94.6 %. For men the model detected 64.5 % of lesions with a much lower specificity of 82 %. The MMT detected 51.2 % of adenomas in women and 44.9 % in men. Consistent with the lower specificity for men, the MMT had a higher sensitivity for cancer among men (98.5 %) than women (82.7 %). The discrepancy appeared to be due to differences in FIT results rather than differences in the microbiome. After correcting for diagnosis, there was a significant effect of sex on FIT result (*p* = 0.006, two-way ANOVA), but not on the overall structure of the microbiome (PERMANOVA: *p* = 0.07). The lower specificity and higher sensitivity for cancer among men is consistent with previous observations that men have a higher positive rate for FIT [34, 35].

We have previously shown that incorporating patient metadata into microbiome-based diagnostic models can improve screening accuracy [17]. To test whether the same was true for the MMT we generated a random forest model that combined patients’ age, BMI, sex, and smoking status with the OTUs and FIT result from the MMT. The AUC of the ROC curve for this model (0.869) was not significantly different from that of the MMT (AUC: 0.829, *p* = 0.11, Additional file 7: Figure S7). When the model with patient metadata was set to the same specificity as the MMT (90.1 %), it did not improve the sensitivity for lesions (63.4 %) compared to MMT (62.9 %, *p* = 0.9). Thus, contrary to our previous findings, incorporation of patient metadata did not significantly improve the MMT.

## Discussion

We confirmed previous findings that the gut microbiota can be used to differentiate healthy individuals from those with colonic lesions. Although FIT was better at detecting cancers than a model using only the microbiota, microbiota-based models detected a subset of lesions that were not detected by FIT. This suggested that the two methods could complement each other. Based on this observation we developed a cross-validated random forest model that combined both FIT and the microbiota to detect colonic lesions. The resulting MMT had higher sensitivity than FIT for detecting lesions, especially adenomas. The MMT was also able to detect the majority of cancers missed by FIT. However, the increased sensitivity of MMT was accompanied by a decrease in specificity compared to FIT. With a false positive rate more than three times higher than FIT (9.9 % versus 2.9 %), an annual MMT would result in more colonoscopies than using FIT as the primary screening test. However, the higher sensitivity of the MMT might make it possible to reduce the frequency of screening, thereby offsetting the difference in the number of colonoscopies. Additional studies would be needed identify the appropriate screening interval and to determine whether the increased number of true positives identified by MMT justify the increased number of false positives.

It was recently shown that when FIT was combined with host-associated DNA biomarkers, the ability to detect adenomas and carcinomas was significantly improved over FIT alone [2]. The sensitivity of the host-associated DNA screen was 92.3 % for cancer and 42.4 % for adenomas with a specificity of 89.8 %, all very similar to what we observed with our MMT. Such results support the assertion that because of the large interpersonal variation in markers for adenomas and carcinomas, it is necessary to employ a panel of biomarkers and to use a model that integrates the biomarkers. The accuracy of our model may be further improved by incorporating additional indicators such as host-associated biomarkers or those targeting specific genes involved in the underlying mechanism of tumorigenesis such as bacterial toxins [15, 16, 18]. More generally, predictive and diagnostic models for other diseases with a microbial etiology may benefit from a similar approach. For example, we recently demonstrated the ability to detect *Clostridium difficile* infection based on the composition of the microbiota [37]. Such models are likely to be useful as microbiota sequencing gains traction as a tool for characterizing health.

Surprisingly most of the OTUs that work well for identifying cancers, including *Fusobacterium nucleatum* (OTU264), *Peptostroptococcus stomatis* (OTU310), and *Parvimonas micra* (OTU281), were excluded from the MMT. This is likely due to these OTUs being positively correlated with FIT (all *p* <0.001, Spearman correlation), meaning they add little information when used in combination with FIT. Instead the MMT is enriched for OTUs that help detect adenomas. Thus the MMT model relies primarily on FIT for detecting cancer, and uses the microbiota to help identify adenomas undetectable by FIT alone. It is also interesting that most of the OTUs used in the MMT were enriched in normal individuals, suggesting that a loss of beneficial organisms in addition to the emergence of pathogens may be important for colorectal cancer development. Many of the OTUs that were depleted in patients with lesions belonged to the Ruminococcoaceae and Lachnospiraceae families, which contain the predominant producers of butyrate, a short-chain fatty acid with anti-inflammatory and anti-tumorigenic properties [38–41]. Likewise Zeller et al. observed a depletion of a potential butyrate-producing *Eubacterium spp.* in patients with CRC [18]. Loss of butyrate or other anti-inflammatory microbial metabolites may contribute to CRC development. These possibilities highlight the need for longitudinal studies to better understand how changes to an individual’s microbiome or the metabolic profile of the gut might predispose them to CRC.

Like other groups, we noticed that the microbiota of CRC patients contained higher levels of bacterial taxa traditionally thought of as oral pathogens, including *Fusobacterium*, *Porphyromonas*, *Peptostreptococus*, *Gemella*, *Parvimonas*, and *Prevotella*. Periodontal pathogens have been shown to promote the progression of oral cancer [42]. Therefore it is possible that these taxa could influence the progression of CRC by a similar mechanism. These observations may warrant further investigation into a potential link between periodontal disease and CRC. Furthermore, since the structure of an individual’s oral microbiome is correlated with that of the gut [43], alterations in the oral community could potentially be a proxy for ongoing or future changes to the gut community.

Although it is exciting that the addition of the microbiota can improve the sensitivity of FIT, further validation is needed prior to clinical adoption. This represents the largest cohort to date, but still only consists of 490 patients. In contrast, the cohort used to validate the Multitarget stool DNA test included 9989 participants. Development of a larger cohort will allow us to apply the MMT to a separate validation set. It is also unclear how sensitive the MMT is to variation in sample preparation and processing. Many of the samples included in the current study were collected 1–2 weeks after the participants’ colonoscopy. A previous study showed that the microbiome quickly returns to normal following colonoscopy [20]. Likewise, we found no difference in the microbiome between samples collected prior to or after colonoscopy (PERMANOVA: *p* = 0.45). Regardless, we would have greater confidence in the predictive potential of the microbiota if all samples were collected prior to colonoscopy. Despite these shortcomings, the ability to improve the sensitivity of detecting adenomas suggests that further methods development and validation are warranted.

## Conclusions

Our findings demonstrate the potential for combining the analysis of a patient’s microbiota with conventional stool-based tests to improve CRC detection. Using the random forest algorithm it was possible to interpret FIT results in the context of the microbiota. The MMT had higher sensitivity for lesions, especially at early stages of tumorigenesis. Moreover the model detected the majority of cancers that FIT was unable to detect. The shortcoming of the MMT is its lower specificity. However, the potential value of the MMT is its higher sensitivity, which is the purpose of preventive screening – finding lesions earlier so that cancer would be avoided.

### Availability of data and materials

Raw fastq files and a MIMARKS file are available through the NCBI Sequence Read Archive (SRP062005). The exact data processing steps for going from the raw sequence data to the final manuscript is available at http://www.github.com/SchlossLab/Baxter_glne007Modeling_GenomeMed_2015.

## Additional files

Additional file 1: Figure S1. Random forest feature selection for detecting adenomas. (A) Change in AUC with varying number of variables in the random forest model. The model with the highest AUC contained 22 OTUs. (B) Importance of each OTU in the model as measured by mean decrease accuracy when the OTU is removed from the model. (C) Relative abundance of the most discriminatory OTUs in adenoma and normal samples. (PDF 19 kb) Additional file 2: Figure S2. Cross-validation of OTU random forest models. ROC curves for the (A) adenoma versus normal OTU model and (B) cancer versus normal OTU model based on OOB estimates, leave-one-out cross-validation, and 10-fold cross-validation. (PDF 13 kb) Additional file 3: Figure S3. Random forest feature selection for detecting cancers. (A) Change in AUC with varying number of variables in the random forest model. The model with the highest AUC contained 34 OTUs. (B) Importance of each OTU in the model as measured by mean decrease accuracy when the OTU is removed from the model. (C) Relative abundance of the most discriminatory OTUs in cancer and normal samples. (PDF 19 kb) Additional file 4: Figure S4. Bacterial OTUs in MMT. (left) Importance of each OTU used in the MMT as measured by the mean decrease in the Gini index when the OTU is removed from the model. (right) Stripchart of the relative abundances of each OTU in the MMT with black lines at the medians. (PDF 76 kb) Additional file 5: Figure S5. Cross-validation of MMT. ROC curves for the MMT model based on OOB estimates, leave-one-out cross-validation, and 10-fold cross-validation. (PDF 9 kb) Additional file 6: Figure S6. MMT performance by sex. ROC curves (left) and stripchart (right) of MMT results separated by sex. (PDF 12 kb) Additional file 7: Figure S7. MMT with patient metadata. ROC curves for distinguishing normal from lesion using FIT, the MMT, or the MMT with metadata. (PDF 8 kb)

## Abbreviations

AUC: area under the curve
CRC: Colorectal Cancer
FIT: fecal immunochemical test
gFOBT: guaic fecal occult blood test
MMT: multitarget microbiota test
OOB: out-of-bag
OTU: operational taxonomic unit
ROC curve: receiver operating characteristic curve
