# Supplementary figures and images for: Microbiota-based model improves the sensitivity of fecal immunochemical test for detecting colonic lesions
Source: Genome Med. 2016 Apr 6;8:37. doi: 10.1186/s13073-016-0290-3 (PMC4823848; doi:10.1186/s13073-016-0290-3)

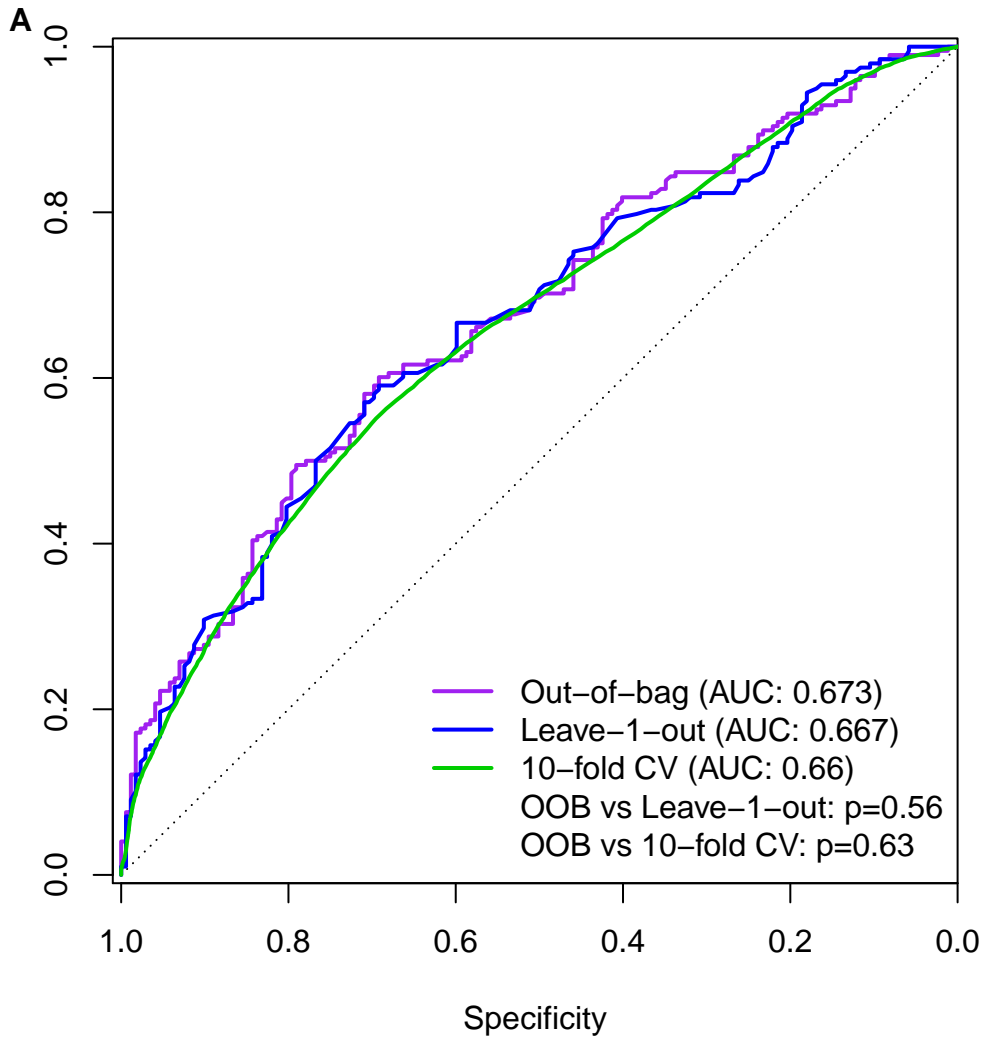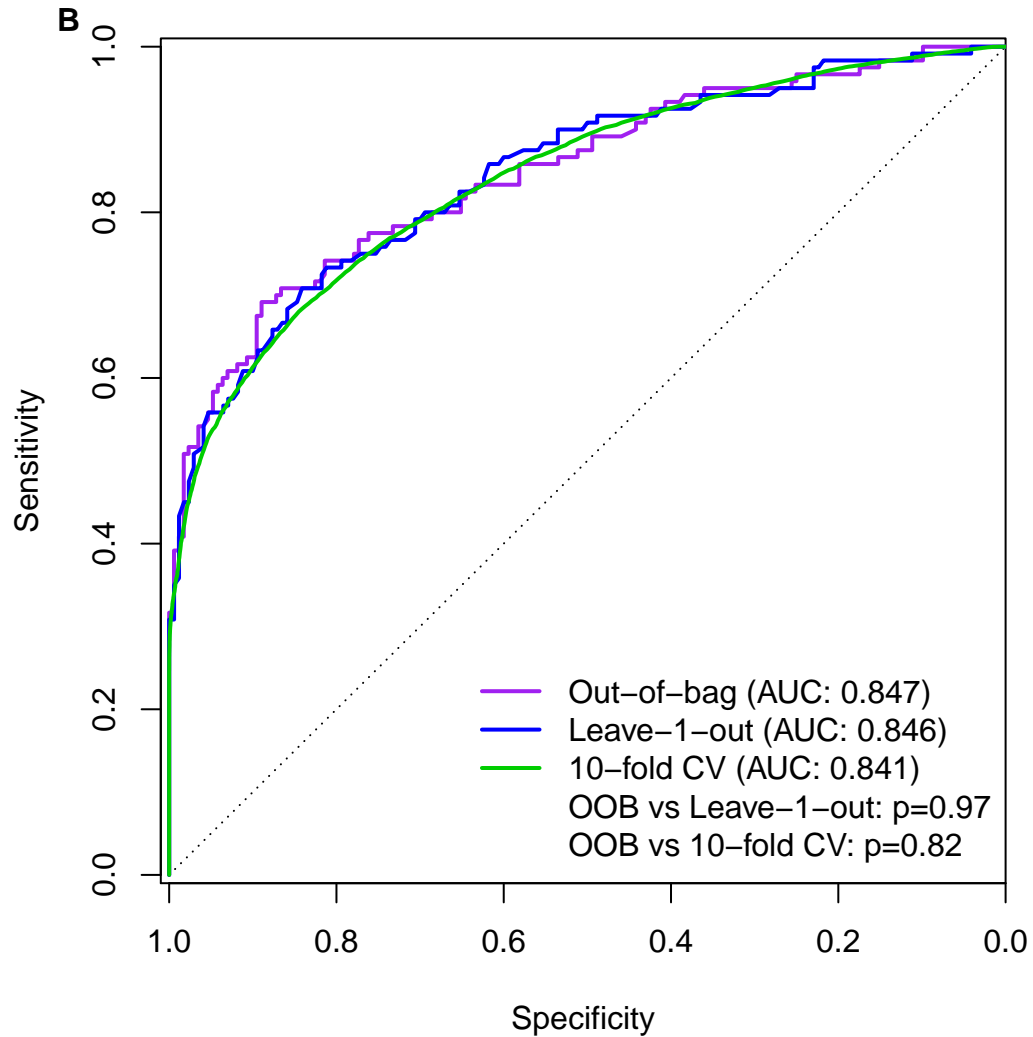

Supplement: Additional file 2: Figure S2. — Cross-validation of OTU random forest models. ROC curves for the (A) adenoma versus normal OTU model and (B) cancer versus normal OTU model based on OOB estimates, leave-one-out cross-validation, and 10-fold cross-validation. (PDF 13 kb) [file 13073_2016_290_MOESM2_ESM.pdf]

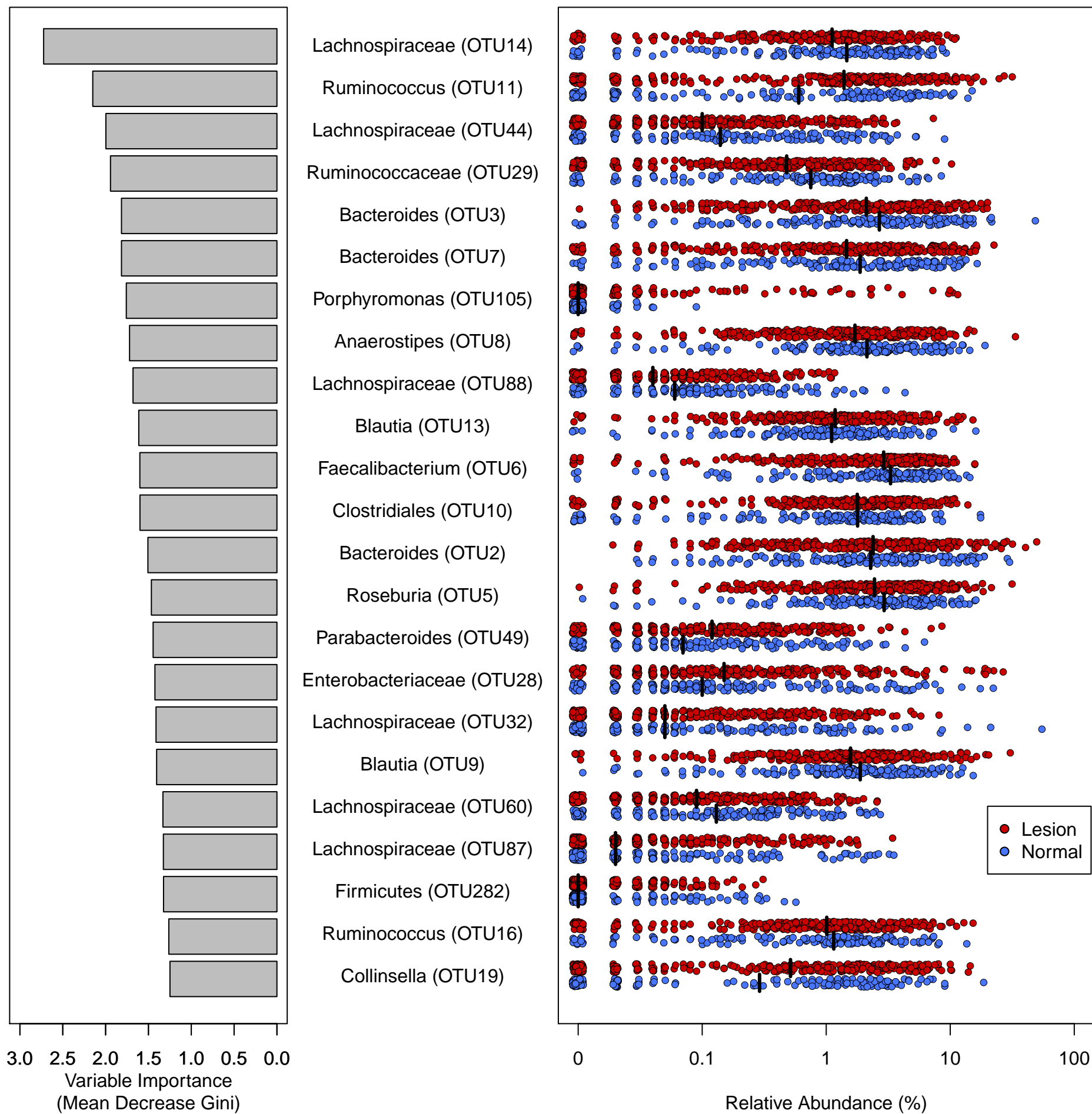

Supplement: Additional file 4: Figure S4. — Bacterial OTUs in MMT. (left) Importance of each OTU used in the MMT as measured by the mean decrease in the Gini index when the OTU is removed from the model. (right) Stripchart of the relative abundances of each OTU in the MMT with black lines at the medians. (PDF 76 kb) [file 13073_2016_290_MOESM4_ESM.pdf]

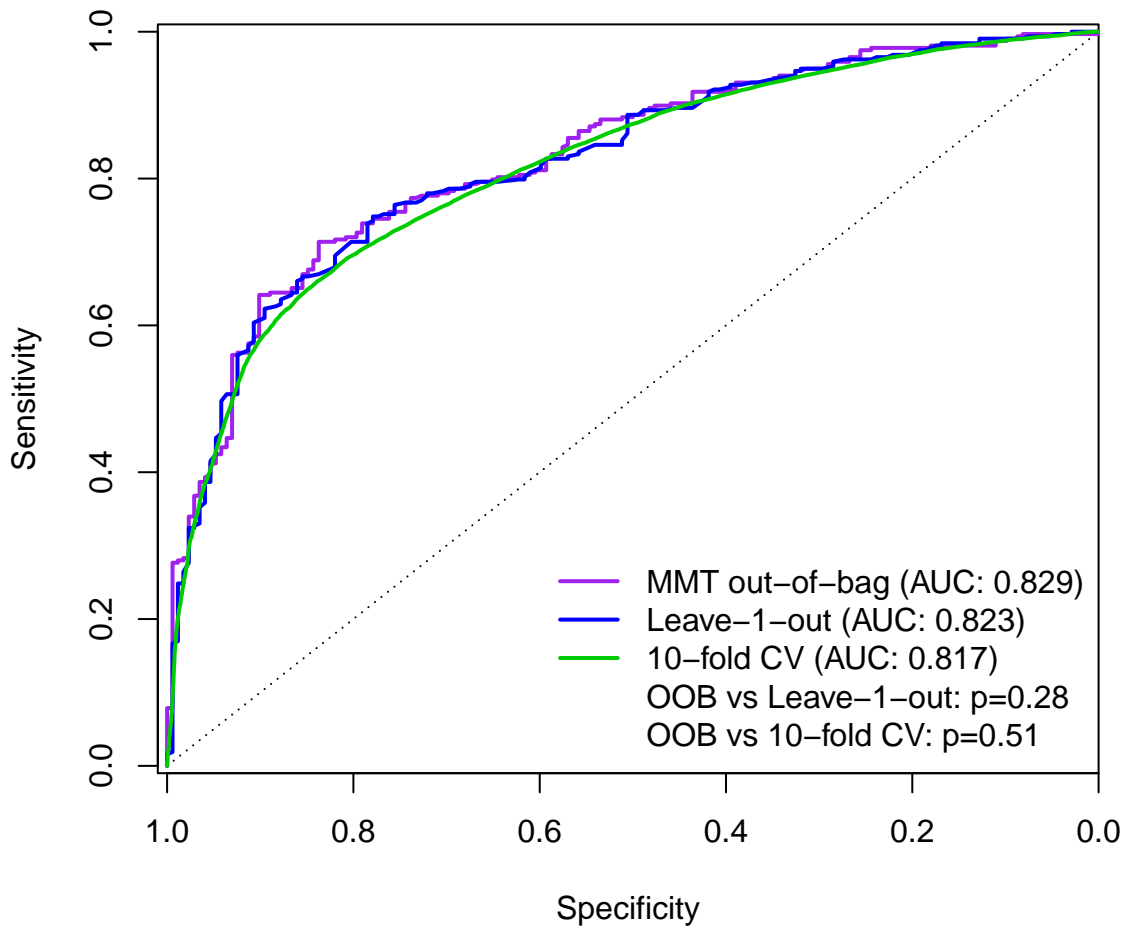

Supplement: Additional file 5: Figure S5. — Cross-validation of MMT. ROC curves for the MMT model based on OOB estimates, leave-one-out cross-validation, and 10-fold cross-validation. (PDF 9 kb) [file 13073_2016_290_MOESM5_ESM.pdf]

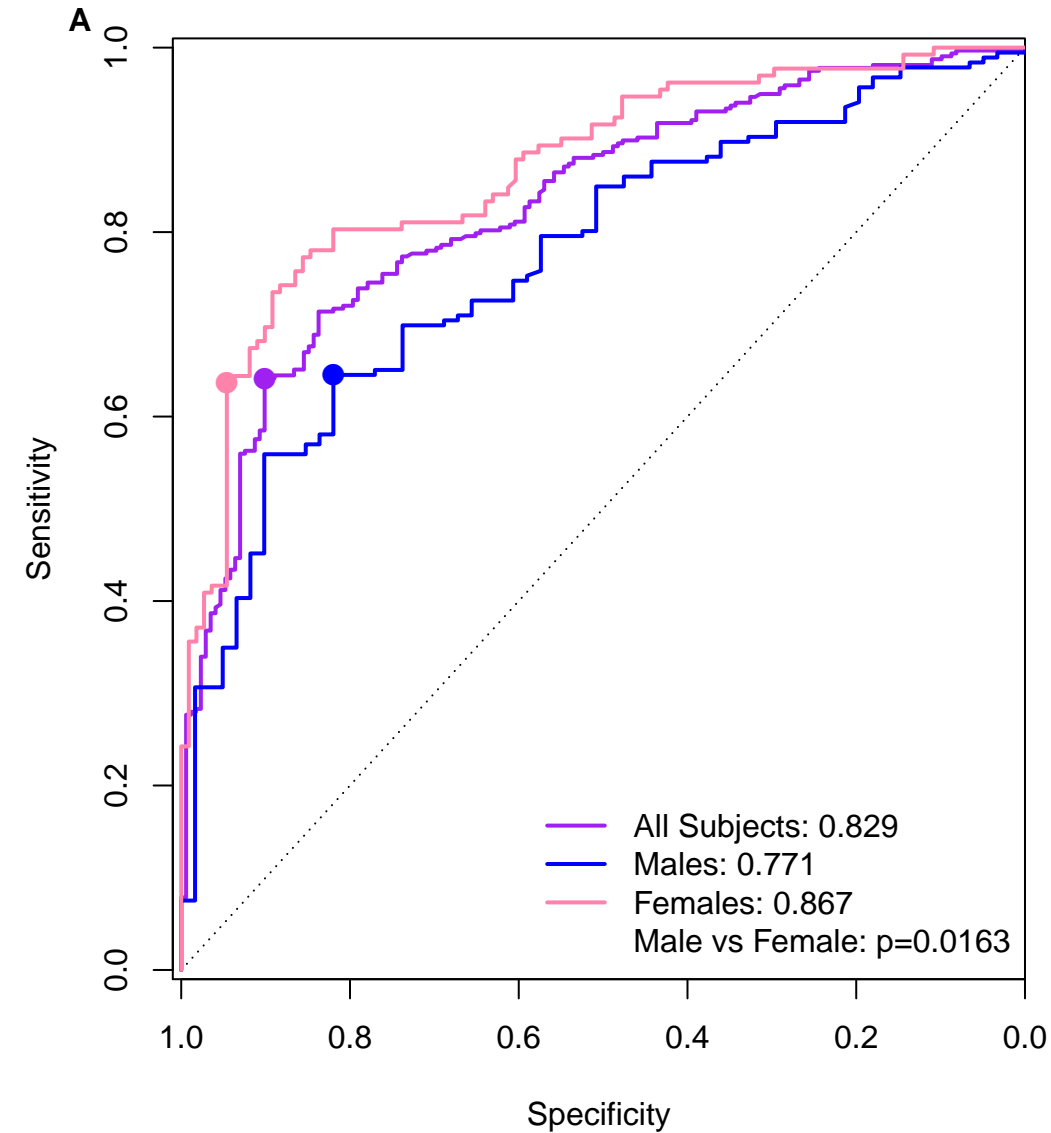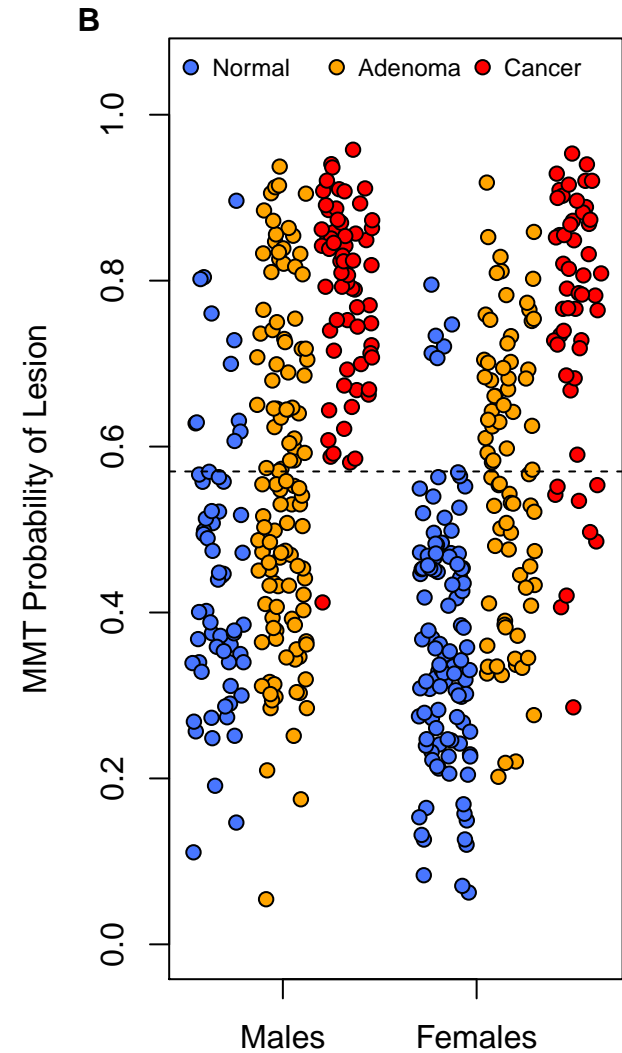

Supplement: Additional file 6: Figure S6. — MMT performance by sex. ROC curves (left) and stripchart (right) of MMT results separated by sex. (PDF 12 kb) [file 13073_2016_290_MOESM6_ESM.pdf]

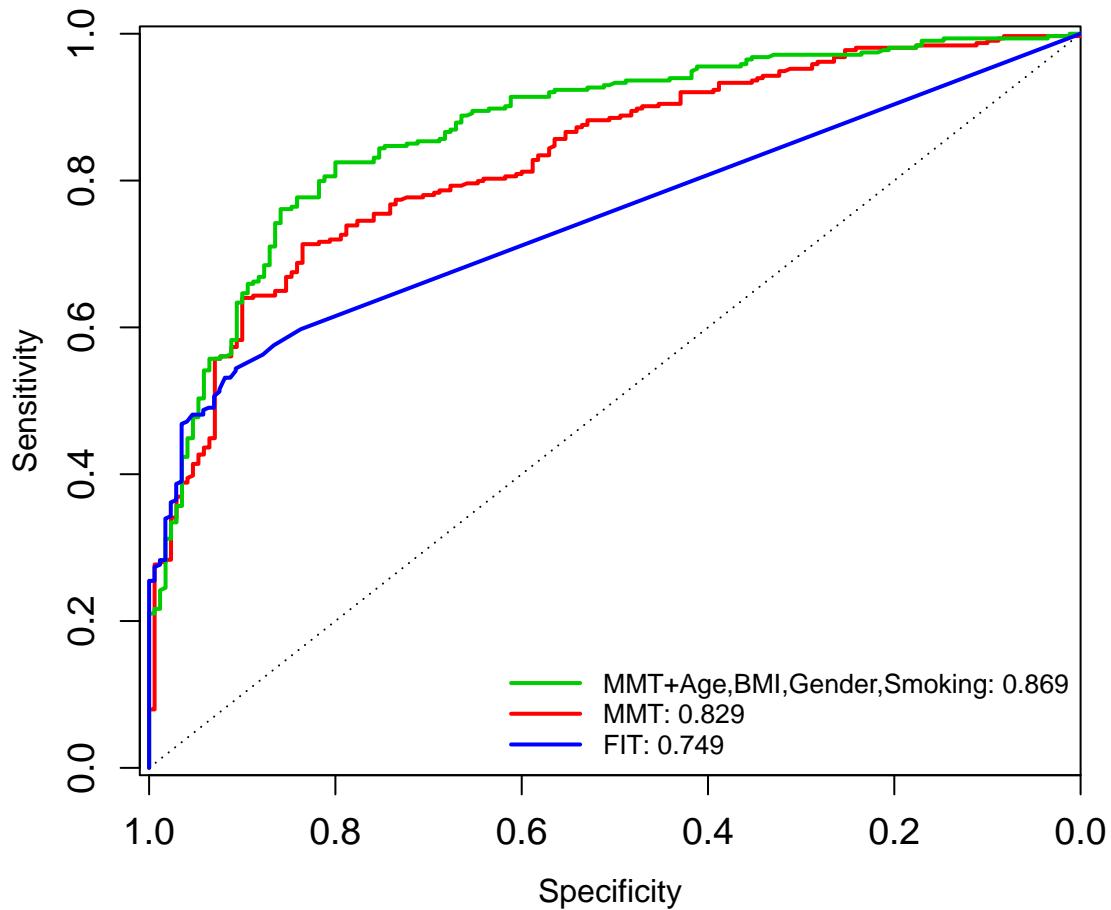

Supplement: Additional file 7: Figure S7. — MMT with patient metadata. ROC curves for distinguishing normal from lesion using FIT, the MMT, or the MMT with metadata. (PDF 8 kb) [file 13073_2016_290_MOESM7_ESM.pdf]
