# Supplementary material for: Microbiota-based model improves the sensitivity of fecal immunochemical test for detecting colonic lesions
Source: Genome Med. 2016 Apr 6;8:37. doi: 10.1186/s13073-016-0290-3 (PMC4823848; doi:10.1186/s13073-016-0290-3)
Supplement: Additional file 3: Figure S3. — Random forest feature selection for detecting cancers. (A) Change in AUC with varying number of variables in the random forest model. The model with the highest AUC contained 34 OTUs. (B) Importance of each OTU in the model as measured by mean decrease accuracy when the OTU is removed from the model. (C) Relative abundance of the most discriminatory OTUs in cancer and normal samples. (PDF 19 kb) [file 13073_2016_290_MOESM3_ESM.pdf]

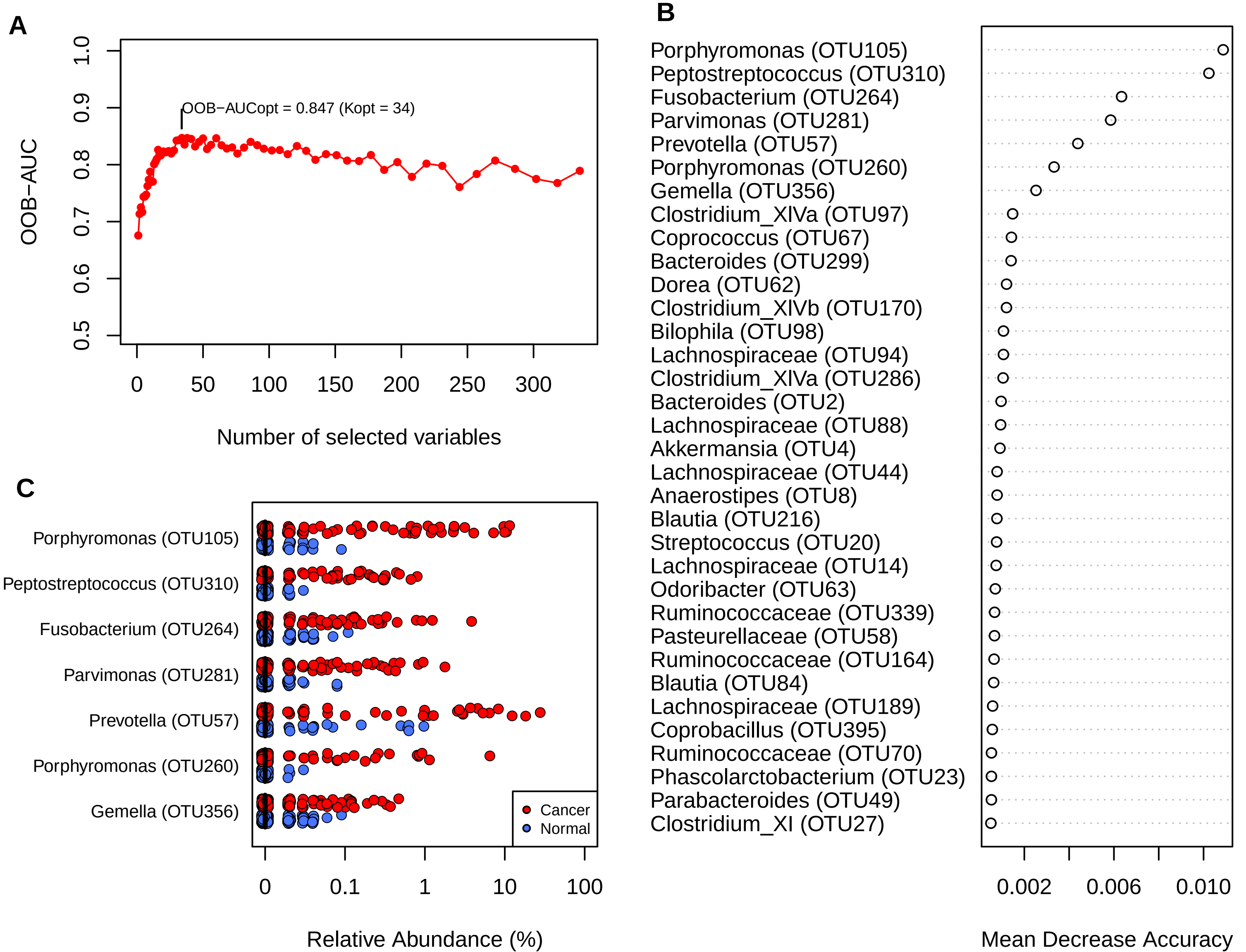
A
OOB−AUC
1.0
0.9
0.8
0.7
0.6
0.5
OOB−AUCopt = 0.847 (Kopt = 34)
0
50
100
150
200
250
300
Number of selected variables
B
Porphyromonas (OTU105)
Peptostreptococcus (OTU310)
Fusobacterium (OTU264)
Parvimonas (OTU281)
Prevotella (OTU57)
Porphyromonas (OTU260)
Gemella (OTU356)
Clostridium_XlVa (OTU97)
Coprococcus (OTU67)
Bacteroides (OTU299)
Dorea (OTU62)
Clostridium_XlVb (OTU170)
Bilophila (OTU98)
Lachnospiraceae (OTU94)
Clostridium_XlVa (OTU286)
Bacteroides (OTU2)
Lachnospiraceae (OTU88)
Akkermansia (OTU4)
Lachnospiraceae (OTU44)
Anaerostipes (OTU8)
Blautia (OTU216)
Streptococcus (OTU20)
Lachnospiraceae (OTU14)
Odoribacter (OTU63)
Ruminococcaceae (OTU339)
Pasteurellaceae (OTU58)
Ruminococcaceae (OTU164)
Blautia (OTU84)
Lachnospiraceae (OTU189)
Coprobacillus (OTU395)
Ruminococcaceae (OTU70)
Phascolarctobacterium (OTU23)
Parabacteroides (OTU49)
Clostridium_XI (OTU27)
0.002
0.006
0.010
Mean Decrease Accuracy
C
Porphyromonas (OTU105)
Peptostreptococcus (OTU310)
Fusobacterium (OTU264)
Parvimonas (OTU281)
Prevotella (OTU57)
Porphyromonas (OTU260)
Gemella (OTU356)
Cancer
Normal
0
0.1
1
10
100
Relative Abundance (%)
